# Anti-Inflammatory and Analgesic Effects of *Schisandra chinensis* Leaf Extracts and Monosodium Iodoacetate-Induced Osteoarthritis in Rats and Acetic Acid-Induced Writhing in Mice

**DOI:** 10.3390/nu14071356

**Published:** 2022-03-24

**Authors:** Yun Mi Lee, Eunjung Son, Seung-Hyung Kim, Dong-Seon Kim

**Affiliations:** 1 KM Science Research Division, Korea Institute of Oriental Medicine, 1672 Yuseong-daero, Yuseong-gu, Daejeon 34054, Korea; candykong@kiom.re.kr (Y.M.L.); ejson@kiom.re.kr (E.S.); 2 Institute of Traditional Medicine and Bioscience, Daejeon University, Daejeon 34520, Korea; sksh518@dju.kr

**Keywords:** inflammation, pain, *Schisandra chinensis* leaf extracts, osteoarthritis

## Abstract

In this study, we aimed to determine the anti-inflammatory and antinociceptive activities of *Schisandra chinensis* leaf extracts (SCLE) in lipopolysaccharide (LPS)-stimulated RAW 264.7 macrophages, an acetic acid-induced mouse model of writhing, and a monosodium iodoacetate (MIA)-induced rat model of osteoarthritis (OA). In LPS-stimulated RAW264.7 cells, a 100 µg/mL dose of SCLE significantly reduced the production of nitric oxide (NO), interleukin-1β (IL-1β), tumour necrosis factor-alpha (TNF-α), interleukin-6 (IL-6), and prostaglandin E2 (PGE2). Acetic acid-induced writhing responses in mice that quantitatively determine pain were significantly inhibited by SCLE treatment. In addition, SCLE significantly decreased the MIA-induced elevation in OA symptoms, the expression levels of pro-inflammatory mediators/cytokines and matrix metalloproteinases, and cartilage damage in the serum and joint tissues. Our data demonstrated that SCLE exerts anti-osteoarthritic effects by regulating inflammation and pain and can be a useful therapeutic candidate against OA.

## 1. Introduction

Osteoarthritis (OA) is a progressive and degenerative disease characterised by the degradation of articular cartilage, changes in the subchondral bone, osteophyte formation, muscle weakness, and inflammation of the synovial membrane; it remains the leading cause of pain and disability in the ageing population [1,2]. Although OA has often been referred to as a non-inflammatory “wear and tear” disease, recent studies have demonstrated that inflammatory mechanisms play an important role in OA pathogenesis [3]. For instance, several studies have shown that the levels of pro-inflammatory mediators increase in the serum and synovial fluid of OA patients, and the pro-inflammatory cytokine stimulation of cartilage tissue induces several structural changes associated with OA [3,4]. Furthermore, patients with OA are characterised by the potential loss of joint cartilage and disruption of bone structure, which can cause pain in the joints [5]. Therefore, inflammation and pain in OA could serve as therapeutic targets for discovering new drugs.

Osteoarthritis treatment aims to improve quality of life by reducing pain, minimising cartilage damage, improving or maintaining functional status, delaying disease progression, and relieving disease [6]. The recommended treatments for OA include aerobic exercise and medication that includes acetaminophen, corticosteroids, or non-steroidal anti-inflammatory drugs to reduce inflammation and pain as well as control the disease. However, these drugs cannot prevent progressive cartilage degradation or repair the damaged cartilage in patients with OA. Moreover, such interventions cannot be used long-term because of their limited efficacy and side effects, including diarrhoea, vomiting, gastrointestinal disturbances, renal toxicity, nausea, and cardiovascular risks [7,8,9]. Despite these challenges, they are widely used to treat OA because of a lack of availability of effective medications. Therefore, the demand for an effective and safe alternative to treat OA has increased significantly. Recent studies have investigated numerous traditional medicinal herbs to develop complementary and alternative approaches, including nutraceuticals, dietary supplements, and healthy functional foods to manage OA, delay disease progression, and reduce pain [9,10].

The fruits of *Schisandra chinensis* (Turcz.) Baill. contain large quantities of dibenzocyclooctadiene lignans, including schizandrol A, schizandrin B, schizandrin C, schizantherin A, schizantherin B, schizandrin A, schisanthenol, and gomisins A and G [11]. *S. chinensis* fruits have been used as medicine for night sweats, dry cough, insomnia, asthma, involuntary ejaculation, urinary disorders, poor memory, chronic diarrhoea, hyperacidity, diabetes, and hepatitis in several countries, including Korea, Russia, and China [12]. Several studies have reported neuroprotective [13], hepatoprotective [14], anti-inflammatory [15], antioxidative [16], and anti-atherosclerotic [17] effects. Additionally, Choi et al. reported that the fruit of *S. chinensis* decreased the production of pro-inflammatory mediators and cytokines and protected articular cartilage degradation in monosodium iodoacetate (MIA)-induced OA of the knee joint in rats [18]. However, most phytochemical and efficacy studies have focused on fruit extracts, particularly lignans. Recently, Liu et al. isolated the lignans terpenoids from *S. chinensis* leaves and demonstrated their potential cytotoxicity effects against MGC-803, Caco-2, and Ishikawa cell lines [19]. However, to the best of our knowledge, the anti-inflammatory and protective effects of *S. chinensis* leaf (SCL) extracts (SCLEs) against OA remain unknown.

In the present study, we investigated the anti-inflammatory and antinociceptive activities of SCLE in vitro against lipopolysaccharide (LPS)-stimulated RAW 264.7, and in vivo using an acetic acid-induced mouse model of writhing and MIA-induced OA in rats.

## 2. Materials and Methods

### 2.1. Preparation of SCLE

*Schisandra chinensis* leaves (100 g) were extracted with 1.5 L of 70% aqueous ethanol for 3 h in a reflux extractor (MS-DM607; M-TOPS, Seoul, Korea). The extracted solution was filtered using a filter paper (Whatman No. 1) and concentrated under reduced pressure in a rotary evaporator (N-1200 A; Eyela, Tokyo, Japan) at 50 °C. After removal of ethanol, the extract was frozen at −80 °C for 2 h and freeze-dried (FDU-2100; Eyela, Tokyo, Japan) for 72 h to obtain a powder (SCLE; yield 22.3%). The extraction process was carried out at a low temperature to minimise oxidisation and degradation of the targeted phenolic compounds.

### 2.2. Chemical Profiling of SCLE

A Waters Acquity Ultra-Performance Liquid Chromatography system equipped with a quaternary pump, auto-sampler, and photodiode array detector (Waters, MA, USA). The chromatographic separation was performed using an Acquity UPLC^®^ BEH C18 (100 × 2.1 mm, 1.7 μm). Each standard stock solution was prepared by dissolving accurately weighed amount of compound in methanol. Quercetin, kaempferol, schizandrol A, gomisin J, schizantherin A, schizandrin A, schizandrin B, and schizandrin C were used after purchasing reference materials (Chromadex, Los Angeles, CA, USA) [19]. An aliquot amount of each stock solution was mixed, and the standard mixture was diluted to a series of appropriate concentrations for the construction of calibration curves. The concentration range (μg/mL) for each calibration curve was as follows: quercetin, 100~500; kaempferol, 100~500; schizandrol A, 10~50; gomisin J, 10~50; schizantherin A, 10~100; schizandrin A, 10~50; schizandrin B, 10~100; schizandrin C, 10~50. An elution with solvent A (water) and solvent B (acetonitrile) in a gradient elution at a flow rate of 0.4 mL/min was carried out as follows: 0–2 min, 20–20% B; 2–6 min, 20–30% B; 6–8 min, 30–35% B; 8–18 min, 35–80% B; 18–20 min, 80–100% B; 20–22 min, 100–20% B; 22–24 min, 20–20% B. The detection wavelength was set to 250 nm. The column temperature was maintained at 40 °C, and the injection volume was 2 µL.

### 2.3. Cell Culture

The murine macrophage cell line RAW264.7 (ATCC, Manassas, VA, USA) was cultured in a complete medium containing Dulbecco’s Modified Eagle Medium (DMEM), 10% heat-inactivated fetal bovine serum, penicillin (100 U/mL), and streptomycin (100 µg/mL) (Gibco Inc., Grand Island, NY, USA) and incubated in 5% CO_2_ at 37 °C. RAW 264.7 cells were seeded on a 96-well plate at a density of 1 × 10^5^ cells/well. The medium was replaced with serum-free DMEM, and 0.5 µg/mL LPS (Sigma-Aldrich Chemical Co., St. Louis, MO, USA) with or without SCLE (50, 100, and 200 µg/mL) was added for 24 h [20].

### 2.4. Cytotoxicity Assay

Cell viability assays were performed to determine the cytotoxicity of SCLE using 3-(4,5-dimethylthiazol-2-yl)-2,5-diphenyltetrazolium bromide (MTT) (Sigma-Aldrich Chemical Co.). MTT was dissolved in serum-free DMEM at 0.5 mg/mL concentration. Then, 100 μL of this solution was added to the cells for 4 h in 96-well culture plates. Cell viability was determined by measuring the absorbance of the coloured MTT formazan crystals formed in each well at a wavelength of 570 nm using a microplate reader and was calculated relative to vehicle controls.

### 2.5. Determination of Nitric Oxide (NO), Prostaglandin E2 (PGE2), and Inflammatory Cytokines Production in the Cells

NO production was analysed as the accumulation of nitrite in culture supernatants using the Griess Reagent System (Promega, Madison, WI, USA). The supernatant was mixed with the same volume of Griess reagent and incubated at room temperature for 5 min. To ensure accurate NO quantification, reference curves were prepared using nitrite standards. The concentration of nitrite was determined by measuring the absorbance using a microplate reader and compared to the nitrite standards reference curve. The serum levels of the pro-inflammatory cytokines, including interleukin-1 beta (IL-1β, MLB00C), interleukin-6 (IL-6, M6000B), tumour necrosis factor-alpha (TNF-α, MTA00B), and prostaglandin E2 (PGE2, KGE004B), were measured using ELISA kits (R&D Systems, Minneapolis, MN, USA), according to the manufacturer’s protocol.

### 2.6. Acetic Acid-Induced Writhing Response in Mice

Institute of Cancer Research (ICR) mice (7 weeks old, 25–30 g body weight) were purchased from Orient Bio (Seongnam, Korea). After acclimation for 1 week, the male ICR mice were randomly assigned to four groups (*n* = 5, each): (1) acetic acid-treated group, (2) SCLE-treated group (150 mg/kg body weight), and (3) SCLE-treated group (300 mg/kg body weight) with acetic acid injection, and (4) ibuprofen (IBU)-treated group (200 mg/kg body weight) with acetic acid injection. After fasting for 14 h, mice were orally administered SCLE (150 and 300 mg/kg body weight) or ibuprofen (IBU, 200 mg/kg body weight). Subsequently, after 1 h, acetic acid (0.75%, 10 µL/g) was injected intraperitoneally [21]. Immediately before use, all samples were dissolved in 0.5% carboxymethyl cellulose (CMC). Five minutes after the acetic acid injection, the number of writhes was recorded for 10 min. The responses included stretching, tension on one side, and the extension of the hind legs. The animal study was approved by the Institutional Animal Care and Use Committee of the Korea Institute of Oriental Medicine (approval number: 20-016).

### 2.7. Development of MIA-Induced OA Rat Model

Seven-week-old male Sprague–Dawley rats were purchased from Orient Bio (Seongnam, Korea). The rats were housed separately in cages for acclimation and familiarised with the testing procedures for 1 week. Afterwards, the rats (8 weeks old) were randomly divided into five groups of seven animals each: (1) control group, (2) MIA group with MIA (3 mg/50 µL 0.9% saline) injection, (3) SCLE-treated group (150 mg/kg body weight), (4) SCLE-treated group (300 mg/kg body weight) with MIA injection, and (5) indomethacin (IM)-treated group (1 mg/kg body weight) with MIA injection. The rats were anaesthetised using a mixture of ketamine (25 mg/0.5 mL) and xylazine (20 mg/0.2 mL). MIA solution was injected into the intra-articular space of the right knee [20,22]. Two millilitres SCLE or IM was dissolved in 0.5% CMC and administered orally for 3 days before MIA injection and then for 21 days. The serum markers of liver damage, serum aspartate aminotransferase (AST) and alanine aminotransferase (ALT), were measured to evaluate the potential toxicity of SCLE. All experiments involving animals were approved by the Institutional Animal Care and Use Committee of Daejeon University and all experiments were performed in accordance with the committee guidelines (Approval No. DJUARB2020-014).

### 2.8. Hind Paw Weight-Bearing Distribution Measurements (Joint Pain Assessment)

The weight-bearing distributions between the OA-induced hind paw and normal hind paw were measured on 0, 7, 14, and 21 days after OA induction using an incapacitance meter (Bioseb Co.; Pinellas Park, FL, USA) [23]. The rats were comfortably placed in a container with their hind paws on two separate sensor plates. When the rat was standing, the weight distribution of both hind paws was naturally adjusted according to the degree of joint pain. The weight distribution ratio was calculated using the following equation: (right weight/[right weight + left weight]) × 100.

### 2.9. Serum Analysis

Blood samples were collected 22 days after OA induction, and then the serum was obtained by centrifugation (3000× *g* for 10 min at 4 °C) and stored at −70 °C until further assays. The levels of IL-1β (Catalogue No. RLB00), IL-6 (R6000B), TNF-α (RTA00), matrix metalloproteinase (MMP)-2 (MMP200), MMP-9 (RMP900), osteocalcin (MBS728975), and deoxypyridinoline (DPD; MBS731799) were measured using ELISA kits (R&D Systems) or MyBioSource (San Diego, CA, USA) according to the manufacturer’s protocol.

### 2.10. Real-Time PCR Analysis

Total RNA was extracted from the articular cartilage tissue using the RNeasy Mini Kit (Qiagen, Hilden, Germany). Total RNA (1 µg) was reverse-transcribed into cDNA using an iScript cDNA synthesis kit (Bio-Rad) according to the manufacturer’s protocol. After cDNA synthesis, quantitative real-time polymerase chain reaction (qRT-PCR) was performed using iQ SYBR Green Supermix (Bio-Rad) and an ABI StepOnePlus™ Real-Time PCR System (Applied Biosystems, Foster City, CA, USA). The sequences of the primers used in this study are shown in Table 1. The samples were denatured at 95 °C for 30 s and then subjected to 40 cycles at 95 °C for 15 s and 60 °C for 60 s. Data were calculated using the ΔΔCt method and expressed as rates of relative abundance of the target genes to GAPDH [24].

### 2.11. Histopathology Analysis

The knee joints were fixed in 10% formalin, decalcified using 20% formic acid, embedded in paraffin. Each specimen was cut into 5 µm thick sections and stained with H&E or Safranin-O-fast green (Sigma-Aldrich Chemical Co.). Histological changes were examined using a light microscope (BX51; Olympus, Tokyo, Japan) and imaged (DP70; Olympus).

### 2.12. Statistical Analysis

All data were presented as the mean ± standard error of the mean. The significant differences were statistically analysed by one-way analysis of variance followed Dunnett’s tests. All analyses were performed using GraphPad Prism 7.0 (GraphPad Software, San Diego, CA, USA). *p*-values less than 0.05 were considered statistically significant.

## 3. Results

### 3.1. Chemical Profiling Analysis of SCLE

**Figure 1:**
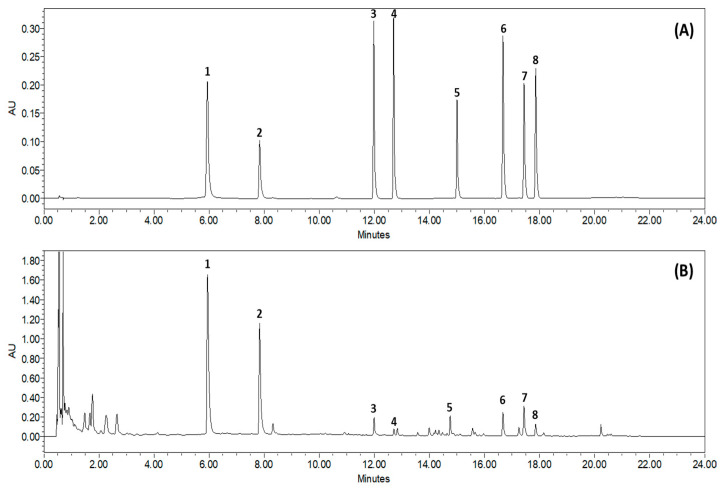
Representative Ultra-Performance Liquid Chromatography chromatogram of *S. chinensis* leaf extracts (SCLE). Mixed standards solution (**A**) and 70% ethanol extract of SCLE (**B**). Quercetin (1), kaempferol (2), schizandrol A (3), gomisin J (4), schizantherin A (5), schizandrin A (6), schizandrin B (7), schizandrin C (8).

Based on the absorption profile and retention time, SCLE was found to contain 70.35 ± 2.617 mg/g quercetin, 96.92 ± 0.612 mg/g kaempferol, 2.76 ± 0.150 mg/g schizandrol A, 0.92 ± 0.055 mg/g gomisin J, 5.98 ± 0.087 mg/g schizantherin A, 5.91 ± 0.441 mg/g schizandrin A, 8.73 ± 0.261 mg/g schizandrin B, and 1.64 ± 0.190 mg/g schizandrin C (Figure 1).

### 3.2. Effect of SCLE on LPS-Induced NO, IL-1β, TNF-α, IL-6, and PGE2 Production In Vitro

**Figure 2:**
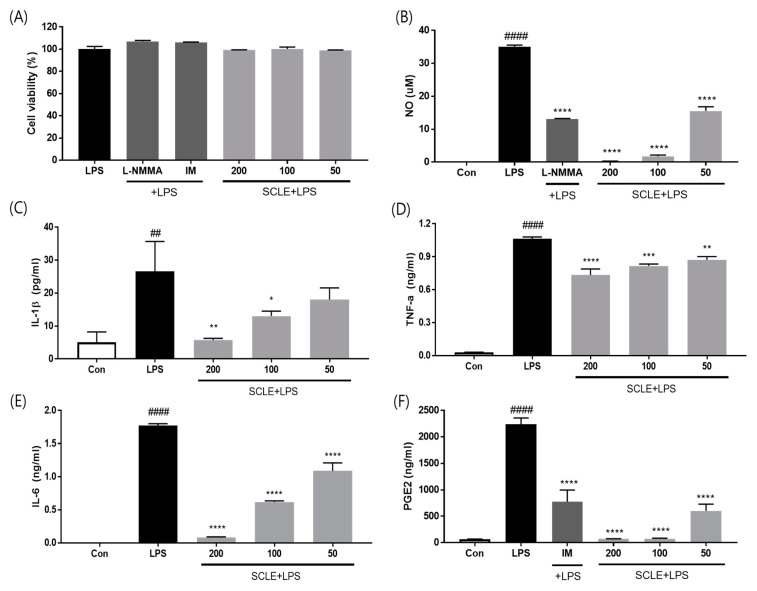
Effect of SCLE on (**A**) cell viability and production of (**B**) NO, (**C**) IL-1β_,_ (**D**) TNF-α, (**E**) IL-6, and (**F**) PGE2 in LPS-treated RAW264.7 cells. NG-methyl-l-arginine (L-NMMA) and indomethacin (IM) were used as positive controls for NO and PGE2 inhibition, respectively. The values are expressed as the mean ± SD (*n* = 3). ^##^ *p* < 0.01, ^####^ *p* < 0.0001 vs. vehicle control cells. * *p* < 0.05; ** *p* < 0.01; *** *p* < 0.001; **** *p* < 0.0001 vs. LPS-treated cells.

To evaluate whether SCLE influences pro-inflammatory cytokine production, LPS-induced RAW264.7 cells were treated with SCLE. SCLE did not affect cell viability (Figure 2A). NO production was measured as the amount of nitrite in LPS-stimulated RAW264.7 supernatant. SCLE remarkably downregulated NO production at doses of 50 (55.7%), 100 (94.8%), and 200 (99.3%) µg/mL (Figure 2B). We also determined the inhibitory effect of SCLE on IL-1β, TNF-α, IL-6, and PGE2 production in LPS-stimulated RAW264.7 cells. LPS remarkably elevated the IL-1β, TNF-α, IL-6, and PGE2 levels, while SCLE significantly reduced the LPS-induced IL-1β (32.1%, 51.0%), TNF-α (23.2%, 31.0%), IL-6 (65.1%, 95.3%), and PGE2 (96.6%, 96.7%) production at 100 and 200 µg/mL, respectively (Figure 2C–F). Positive controls for NO and PGE2 inhibition were NG-methyl-l-arginine (L-NMMA) and indomethacin (IM), respectively.

### 3.3. Anti-Analgesic Effect of SCLE on the Acetic Acid-Induced Writhing Response in Mice

**Figure 3:**
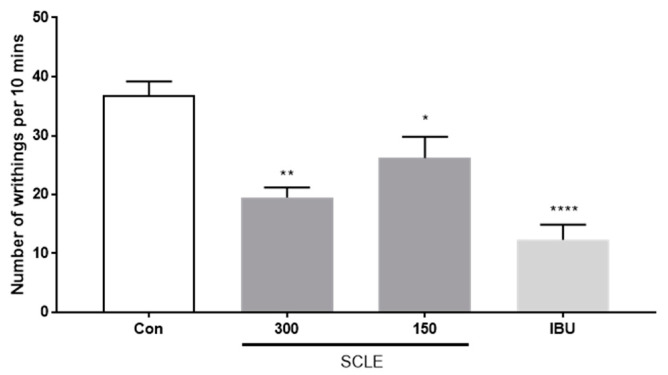
Effect of SCLE on writhing response in acetic acid-induced mice. The writhing number was measured after 0.75% acetic acid injection for 10 min. *n* = 5 per group. * *p* < 0.05, ** *p* < 0.01 and **** *p* < 0.0001 vs. control.

As shown in Figure 3, SCLE at 150 and 300 mg/kg showed 28.8 and 47.0% inhibition of writhing, respectively, as a percentage of the control group 10 min after acetic acid injection. Positive control, ibuprofen (IBU, 200 mg/kg), reduced writhing response by 66.6%. These data indicated that SCLE has an antinociceptive effect on acetic acid-induced writhing response.

### 3.4. Effect of SCLE on Joint Pain in MIA-Induced Osteoarthritis in Rats

**Figure 4:**
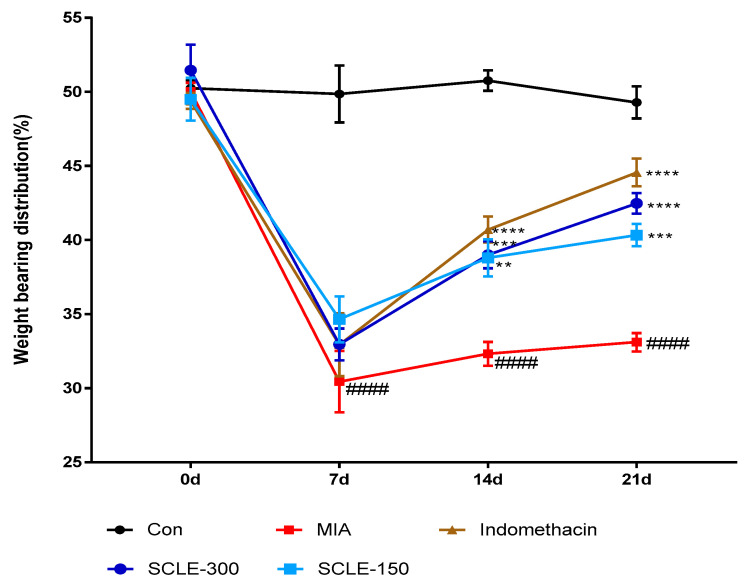
Effects of SCLE on changes in the hind paw weight-bearing distribution in monosodium iodoacetate (MIA)-induced osteoarthritis (OA) rats. The treatment effects of SCLE were compared with that of the MIA-induced OA group. *n* = 7 per group. ^####^ *p* < 0.0001 vs. control; ** *p* < 0.01, *** *p* < 0.001, and **** *p* < 0.0001 vs. MIA-treated rats.

For joint pain, weight distribution was measured in the sensitised and contralateral hind paws to assess OA progression. The weight-bearing distribution of the MIA group decreased rapidly. It became significantly different from that of the control group on day 7 post-MIA injection, and this difference was maintained for at least 21 days. These values were increased slightly in the SCLE- and IM-treated groups on day 7 compared with that in the MIA group. However, these groups restored and balanced between both hind legs after 14 days. SCLE at 150 and 300 mg/kg and IM groups reduced maximum pain values of 36.7, 49.3, and 62.7% at 21 days after MIA induction, respectively. These results demonstrated the significant recovery of hind paw weight-bearing in the SCLE-treated groups (Figure 4).

### 3.5. Effect of SCLE on Inflammatory Mediators and Cytokines in MIA-Induced Rats

**Figure 5:**
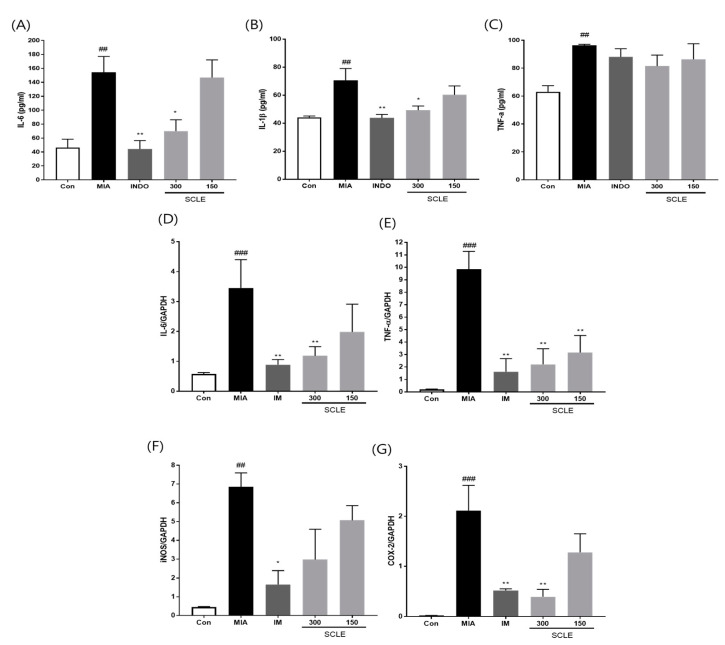
Effects of SCLE on the inflammatory cytokines and mediators in serum and joint tissue of monosodium iodoacetate (MIA)-induced osteoarthritis (OA) rats. (**A**) Serum IL-6, (**B**) IL-1β, and (**C**) TNF-α levels. (**D**) IL-6, (**E**) TNF-α, (**F**) iNOS, and (**G**) COX-2 expression. *n* = 7 per group. ^##^ *p* < 0.01, ^###^ *p* < 0.001 vs. control; * *p* < 0.05; ** *p* < 0.01 vs. MIA-treated rats.

Because inflammation is a critical factor associated with OA pathogenesis [25], we evaluated the effect of SCLE on the production of pro-inflammatory cytokines and inflammatory mediators in MIA-induced rats. The MIA group elevated serum levels of IL-6, IL-1*β*, and TNF-α compared with those in the control group (*p* < 0.01). In contrast, the serum levels of IL-6 and IL-1*β* were remarkably lower in OA rats treated with IM (*p* < 0.01, *p* < 0.01) and 300 mg/kg of SCLE (*p* < 0.05, *p* < 0.05) than in MIA-induced OA rats. Additionally, serum TNF-α levels decreased following SCLE treatment; however, this difference was not significant (Figure 5A–C). We next determined the effect of SCLE on the mRNA expression levels of the inflammatory mediators TNF-α, IL-6, iNOS, and COX-2, in the knee joint tissues of MIA-induced OA rats. The mRNA expression of TNF-α, IL-6, iNOS, and COX-2 increased significantly in the MIA group compared to the control group (Figure 5D–G). However, the expression levels of these genes were significantly suppressed in the IM and 300 mg/kg SCLE groups. These data suggested that SCLE inhibits inflammatory responses by reducing inflammatory mediators and cytokines in MIA-induced OA rats.

### 3.6. Effect of SCLE on Matrix Degradation and Bone Metabolism in MIA-Induced Rats

**Figure 6:**
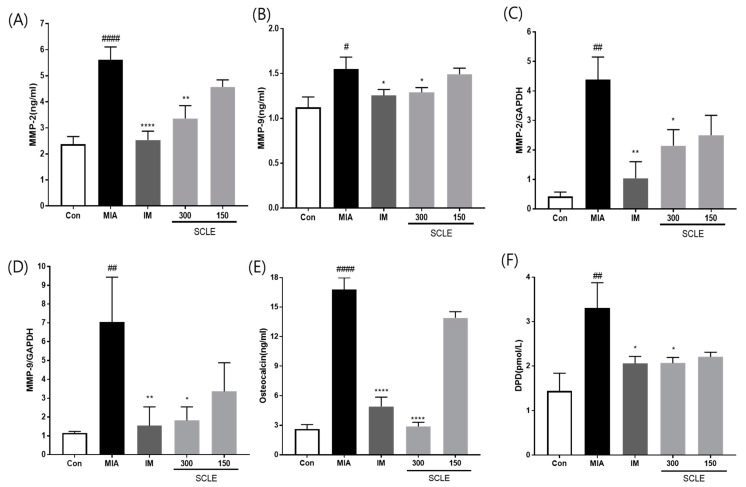
Effects of SCLE on cartilage degradation and bone metabolism in monosodium iodoacetate (MIA)-induced rats. The levels of (**A**) MMP-2, (**B**) MMP-9 in serum and the mRNA expression levels of (**C**) MMP-2 and (**D**) MMP-9 in knee joint tissues. The levels of (**E**) osteocalcin, and (**F**) DPD in serum. *n* = 7 per group. ^#^ *p* < 0.05, ^##^ *p* < 0.01, ^####^ *p* < 0.0001 vs. control; * *p* < 0.05; ** *p* < 0.01, **** *p* < 0.0001 vs. MIA-treated rats.

MMPs are secreted by cells and promoted by inflammatory mediators during the inflammatory process [26]. We determined the effect of SCLE on matrix degrading enzymes, MMP-2 and MMP-9 using ELISA in serum and mRNA expression in knee joint tissues from MIA-induced OA rats. MMP-2 and MMP-9 levels were significantly elevated in the MIA group compared with those in the control group in both serum and knee joint tissues (Figure 6A–D). However, these levels were remarkably suppressed in the IM and 300 mg/kg SCLE-treated groups. To further investigate the effects of SCLE on bone metabolism, we measured the levels of DPD, a marker of bone resorption, and osteocalcin, a biochemical marker of bone formation. As shown in Figure 6E,F, our results indicated a significant increase in osteocalcin and DPD levels in MIA-treated rats, which indicated an imbalance in bone metabolism [18]. In contrast, SCLE significantly decreased the MIA-induced increase in marker levels. Considered together, these results suggested that SCLE may have inhibitory effects on cartilage degradation and bone metabolism in MIA-induced rats.

### 3.7. Effect of SCLE on Articular Cartilage Damage in MIA-Induced Rats

**Figure 7:**
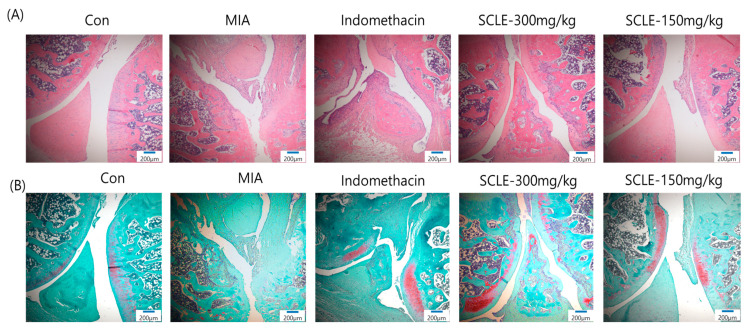
Histopathological features of knee joint tissues in monosodium iodoacetate (MIA)-induced osteoarthritis (OA) rats. Knee joint tissues histopathological changes with (**A**) H&E and (**B**) Safranin-O/Fast Green (magnification, 100×).

Histopathological analysis using H&E staining revealed that the MIA group showed cartilage degeneration, an irregular articular cartilage surface, and a greater increase in inflammatory cell infiltration in the synovial membrane and cartilage of the MIA-treated joint than that in the control joint. In contrast, histological damage and cartilage degeneration in the SCLE- and IM-treated groups had reduced, the articular cartilage surface was less irregular, and inflammatory cell infiltration was prominent (Figure 7A). In addition, we stained the proteoglycan layer and cartilage cells with Safranin-O to evaluate cartilage degradation. The red-stained control cartilage was destroyed by MIA, and the proteoglycan layer disappeared in the MIA-treated group. However, these histomorphological changes were decreased in the SCLE- and IM-treated rats (Figure 7B).

## 4. Discussion

The fruit of *Schisandra chinensis* (Turcz.) Baill. (SC) is a well-known traditional medicine used to treat spontaneous sweating, chronic cough, asthma, and palpitations. Recent studies reported that SC attenuates the inflammatory response in macrophage and chondrocytes [15,27]. Additionally, SC decreased the production of pro-inflammatory cytokines and protected cartilage from degradation in an MIA-induced OA rat model [18]. However, no study has investigated the effectiveness of SCLE in relieving inflammation and OA. This study demonstrated that SCLE had anti-inflammatory effects in LPS-induced RAW 264.7 cells and the MIA-induced OA rat model. We investigated the antinociceptive effects on weight-bearing distribution in MIA-induced OA rats and writhing in acetic acid-induced mice.

OA is characterised by cartilage degradation, synovitis, osteophyte formation, and subchondral bone changes, the most prevalent of which is pain associated with inflammation [28]. Inflammation is a key factor associated with cartilage matrix degradation, and the presence of inflammatory cytokines may cause the loss of articular cartilage homeostasis via metabolic changes and markedly accelerate joint damage. High levels of pro-inflammatory cytokines such as IL-1β, IL-6, IL-10, and TNF-α have been found in several experimental animal models of cartilage degradation and the synovial fluid of OA patients. Among these cytokines, IL-6, IL-1β, and TNF-α are important in OA pathogenesis and disease severity [29]. IL-6, whose production is stimulated in response to TNF-α and IL-1β in diseased joint tissues, is considered a major cytokine that causes changes in the subchondral bone layer. IL-6 promotes osteoclasts formation and thus bone resorption while exhibiting a synergistic effect with TNF-α and IL-1β. Furthermore, TNF-α blocks the chondrocyte synthesis of proteoglycans and type II collagen. IL-1β activates transcription factors via the MAPK and NF-κB signalling pathways to regulate inflammatory responses, resulting in the production of inflammatory mediators such as NO, PGE2, COX-2, and other catabolic factors that may accelerate cartilage degeneration [30,31,32]. Moreover, cytokines upregulate the gene expression of MMPs, which promotes the degradation of articular cartilage by breaking down proteoglycan, the main component of the ECM [33,34]. MMP-2 and MMP-9 are gelatinases that degrade a broad range of collagen and proteoglycans. Therefore, inhibition of IL-6, IL-1β, and TNF-α upregulation might prevent the degradation of joint cartilage during the pathogenesis of OA.

Lipopolysaccharide (LPS)-treated RAW 264.7, the most commonly used model for in vitro studies on inflammatory responses, releases various inflammatory mediators, cytokines, and chemokines, among others, via the activation of inflammatory pathways [35]. In addition, MIA disrupts glycolytic energy metabolism in chondrocytes and causes cell death, leading to inflammation and cartilage damage [22]. Our findings showed that SCLE induced the downregulation of IL-6, IL-1β, and TNF-α production not only in RAW 264.7 but also in MIA-induced OA rat serum and knee joint tissues. In addition, SCLE significantly suppressed the mRNA expression levels of matrix-degrading enzymes, MMP-2 and MMP-9, and inflammatory mediators, iNOS and COX-2, in the knee joint tissue. The histopathological analysis showed that SCLE and IM treatment improved cartilage thickness and conditions compared with those in MIA-induced rats, which led to reduced cartilage destruction. Increased levels of DPD, an indicator of increased bone resorption, and osteocalcin, a biochemical indicator of bone formation in MIA-induced rats, were significantly inhibited with SCLE administration. Collectively, these observations suggested that SCLE alleviates inflammation and cartilage damage modulated via the regulation of the production of pro-inflammatory cytokines and mediators, MMP activity, and histopathological changes.

The incapacitance test, which measures hind paw weight-bearing capacity, is a representative behavioural test useful and relevant in joint pain studies, including the MIA-induced OA model [36]. The weight-bearing capacity of SCLE-treated rats, was superior to that of the MIA group rats and was significantly higher than that of the positive control IM-treated group. The improved weight-bearing capacity of hind paws induced by SCLE in rats may provide pain relief in OA of the knee, which is currently the main goal of OA treatment. These results suggested that SCLE has analgesic effects against OA.

Clinical evidence has suggested that OA pain is driven predominantly by peripheral inputs, as joint treatment or replacement with peripheral analgesics can relieve the OA pain in most cases [37]. The acetic acid-induced writhing test was performed to assess the analgesic effects of SCLE on peripheral pain. Reportedly, acetic acid injection into the peritoneal cavity induces pain by releasing pain mediators, which leads to a writhing response in animals [38]. In this study, SCLE showed high efficacy in suppressing peripheral pain by inhibiting the acetic acid-induced writhing response in mice in a dose-dependent manner. Taken together, these results suggested that the peripheral analgesic effect of SCLE contributes to pain relief in MIA-induced OA.

Kaempferol and quercetin, the main ingredients in SCLE, and other minor ingredients, such as dibenzocyclooctadiene lignans, including schizandrin A, B, and C, are known to have anti-inflammatory effects in articular chondrocytes and chondroprotective effects [39,40,41,42,43]. In addition, numerous other compounds are found in SCLE. Considering these reports of representative compounds, it can be inferred that the therapeutic effects of SCLE against OA could be due to the synergistic anti-inflammatory and chondroprotective effects of these compounds. In this study, we observed the anti-inflammatory and pain-relieving effects of SCLE in LPS-stimulated RAW264.7, MIA-induced OA rats, and acetic acid-induced writhing in mice.

## 5. Conclusions

The present study demonstrated that SCLE can relieve pain and reverse cartilage degeneration by inhibiting the inflammatory responses. The findings suggested that SCLE is a potential candidate for relieving inflammation and pain in OA patients. Further study is needed to evaluate the active ingredients of SCLE responsible for the therapeutic activities and elucidate their mechanisms of action and the signalling pathways involved.

## Figures and Tables

**Table 1:** Sequences of the primers used for the real-time PCR.

Gene		Primer Sequence
IL-1β	Forward	5′-ACAAGGCTGCCCCGACTA T-3′
	Reverse	5′-CTCCTGGTATGAAGTGGCAAATC-3′
IL-6	Forward	5′-GCC CTT CAG GAA CAG CTA TGA-3′
	Reverse	5′-TGTCAACAACATCAGTCCCAAGA-3′
COX-2	Forward	5′-CCTCGTCCAGATGCTATCTTTG-3′
	Reverse	5′-GAAGGTCGTAGGTTTCCAGTATT-3′
iNOS	Forward	5′-CTTTACGCCACTAACAGTGGCA-3′
	Reverse	5′-AGTCATGCTTCCCATCGCTC-3′
MMP-2	Forward	5′-CACCAAGAACTTCCGACTATCC-3′
	Reverse	5′-TCCAGTACCAGTGTCAGTATCA-3′
MMP-9	Forward	5′-CCCAACCTTTACCAGCTACTC-3′
	Reverse	5′-GTCAGAACCGACCCTACAAAG-3′
GAPDH	Forward	5′-TGGCCTCCAAGGAGTAAGAAAC-3′
	Reverse	5′-CAG CAA CTG AGG GCC TCT CT-3′

## Data Availability

The data presented in this study are available in this article.
